# Electrochemical Fingerprint of Arsenic (III) by Using Hybrid Nanocomposite-Based Platforms

**DOI:** 10.3390/s19102279

**Published:** 2019-05-17

**Authors:** Gheorghe Melinte, Oana Hosu, Mariagrazia Lettieri, Cecilia Cristea, Giovanna Marrazza

**Affiliations:** 1 Department of Analytical Chemistry, Faculty of Pharmacy, “Iuliu Haţieganu” University of Medicine and Pharmacy, Pasteur 4, 400349 Cluj-Napoca, Romania; melintegheorghe@gmail.com (G.M.); hosuoanaalexandra@gmail.com (O.H.); ccristea@umfcluj.ro (C.C.); 2 Department of Chemistry ”Ugo Schiff”, University of Florence, Via della Lastruccia 3, 50019 Sesto Fiorentino (Fi), Italy; mariagrazia.lettieri@unifi.it; 3 Instituto Nazionale Biostrutture e Biosistemi (INBB), Unit of Florence, Viale delle Medaglie d’Oro 305, 00136 Roma, Italy

**Keywords:** electrochemical sensor, nanoparticles, screen-printed electrochemical cell, arsenic

## Abstract

Arsenic, one of the most abundant mineral and also one to the most toxic compounds. Due to its high toxicity sensitive analytical methods are highly important, taking into account that the admitted level is in the range of µg L^−1^. A novel and easy to use platform for As(III) detection from water samples is proposed, based on gold and platinum bi metallic nanoparticles and a conductive polymer (polyaniline). The electrochemical detection was achieved after optimization of cathodic pre-concentration and stripping parameters by square wave anodic stripping voltammetry at modified screen-printed carbon-based electrochemical cells, proving its applicability for disposable and cost-effective in situ analysis of arsenic.

## 1. Introduction

Arsenic (As) is the 12th most abundant mineral in the human body and one of the most 20 abundant minerals in the Earth’s crust [1]. It is considered one of the most toxic compounds having a high carcinogenic potential. The exposure to arsenic can cause DNA mutation which can lead to an aberrant gene expression and carcinogenesis [2]. Many regions around the world are affected by arsenic contamination; it appears mostly in areas like Argentina, Bangladesh, China, India, Nepal, Mexico, Pakistan, and parts of the USA. In some waters in Bangladesh, the arsenic level can reach up to 2500 µg L^−1^ while the WHO admitted level is only 10 µg L^−1^ [3]. Moreover, recent studies showed that arsenic contamination is a public health menace in several Italian areas as high levels of arsenic (up to 295 µg L^−1^) can be found in groundwater due to the volcanic origins of the territory [4,5]. Furthermore, arsenic values in drinking water were chronically detected between 20 and 50 μg L^−1^ [4]. As regulations from the EU were delayed for several years to implement more controlled water content, the inhabitants were subjected to long exposure of arsenic at low-medium levels.

It appears in the atmosphere mostly as As(III) and As(V) inorganic salts, and it is usually converting one form to another in solution [6]. Arsenic’s adverse effects are strongly related with the dose, species, and the duration of exposure. A fast and high-level exposure leads to acute effects like nausea, diarrhea, abdominal pain or death. If the exposure is made slow and with low amounts of arsenic, chronic effects will occur, like skin irritation, cardiovascular events, or even cancer, due to its carcinogenic potential [7]. 

Hence, analytical techniques capable of detecting trace levels of As are highly required. Currently used analytical methods for arsenic detection include atomic absorption and emission spectrometry, mass spectrometry, and high-performance liquid chromatography [8]. Although these methods present good limits of detection (LOD) and high sensitivities, several limitations should be mentioned: the need of complex equipment and skilled personnel, complicated sample preparation, time consuming analysis, and most important the poor suitability for in situ detection and routine analysis. 

Electrochemical sensors have been widely applied to sensitively detect a plethora of chemical and biological analytes. The use of electrochemical platform has advantages as multiplexing, portability, cost-effectiveness, and friendly use. In the last decade electrochemical sensors proved to be promising alternatives to qualitatively and quantitatively detect arsenic from different matrices. Different electrochemical methods have been commonly used such as linear sweep anodic stripping voltammetry (LSASV), square wave anodic stripping voltammetry (SWASV), differential pulse anodic stripping voltammetry (DPASV), cathodic stripping voltammetry (CSV), cyclic voltammetry (CV), and chronopotentiometry [9,10]. The methods involving a potential-assisted electrochemical pre-concentration prior the stripping voltammetry revealed to be more sensitive towards As and continues to be one of the most powerful techniques for its electrochemical determination. As(III) is electrochemically reduced to As(0) at the surface of working electrode by cathodic pre-concentration deposition at a controlled time (*t_dep_*) and potential (*E_dep_*) [11]. Therefore, the optimization of these parameters is of crucial importance to obtain the sensor with the best analytical performance. Following the pre-concentration step, the anodic stripping scan is performed to achieve the re-oxidation of As(0) to As(III). However, oftentimes long accumulation period is required and poor selectivity is reported when applied in complex matrices [11]. Therefore, different strategies have been followed in order to increase the sensitivity and the selectivity of electrochemical sensors. Generally, the integration of nanocomposite materials in sensors’ development leads to increased overall analytical performance, while selectivity is provided by the use of biomolecules that specifically react with the target analyte [12].

A great deal of progress has been made in the electrochemical detection of arsenic. One of the first developed electrochemical sensors was based on the co-precipitation of arsenic with copper and selenium followed by its reduction to arsine by means of a mercury drop electrode. The sensor had a low LOD of 0.52 µg L^−1^, but the selectivity was not at a high level, Cu(II) being an important interferent. Even more, the experimental conditions are highly toxic and unlikely to be applied for in situ determination [13]. As the years passed, new types of electrodes and platforms were developed, while nanoparticles gained more and more attention. Gold nanoparticles (AuNPs) has been attracting interest for As detection due to their high adsorption capacity and abundant availability [10]. In 2008, Rassaei et al. developed a sensor for As(III) water detection using a boron-doped diamond electrode and AuNPs. Even though the method was proved to be sensitive as a 1 µg L^−1^ limit of detection was achieved, the electrochemical detection method requires relatively long accumulation time (300 s) of As at the modified-electrode surface [14]. Recent years showed an increase in the number of chemical sensors based on different nanoparticles and nanomaterials for arsenic determination. Carbon electrodes were modified with different nanoparticles obtained from metals such as Mn, Co, Fe, Sn, Au, Pt each one with its advantages and disadvantages. A series of authors report arsenic electrochemical detection using glassy carbon electrodes (GCE) modified with AuNPs and different other nanomaterials [8]. Chowdhury et al. developed a AuNP/polyaniline sensor, that offered a LOD of 0.4 µg L^−1^ for arsenic [15]. Electrodeposited PtNPs at GCEs lead to a LOD for arsenic using LSV of 0.05 µg L^−1^ [16]. Even though there are many metallic nanoparticles based electrochemical sensors for arsenic, with good analytical performance, the most used immobilization electrode (GCE) increases the detection time and does not offers the possibility for in situ detection, like the screen-printed electrochemical cells (SPE) do. As bioelements, aptamers and DNA proved to be extremely selective, but require long preparation time for sensors [17]. By contrast, nanoparticles have the ability to enhance the sensitivity by increasing the active surface of the electrodes due to their electronic, chemical, and physical properties, leading to low detection limits and improved signal-to noise ratios [18]. Several types of metallic nanoparticles are currently used for single or array of electrodes, presenting the significant advantage of being prepared by the electrochemical method starting from the corresponding metallic salts. 

Recently, bimetallic NPs were integrated into sensor design, due to their improved electrocatalytic activity and conductivity. They (i.e., Au-Ag, Au-Pt, Au-Cu, etc.) could be easily integrated in different types of matrices for 3D porous networks capable of detecting heavy metals or other pollutants [8].

In this paper, a sensitive sensor for arsenic detection in water samples is presented based on a mixed film of polyaniline (PANI) and bimetallic composite Au-PtNPs. PANI is one of the most promising conducting polymers. Between its advantages, the fast synthesis, low cost and high electrochemical conductivity are the most important. Nevertheless there are some drawbacks of the PANI that limit its application like low power density and cycling stability [17,19,20]. To overcome these limitation other materials are often incorporated with PANI in the synthesis of novel electrochemical platforms [21]. Different strategies were evaluated in order to obtain the sensor with the best analytical performance for As sensing, namely modified screen-printed carbon electrode (SPCE) with different nanostructured surfaces: gold nanoparticles (AuNPs/AuSPCE), platinum nanoparticles (PtNPs/SPCE), bimetallic (Au-PtNPs/SPCE), polymer (PANI/SPCE), and composite (Au-PtNPs/PANI/SPCE). Hence, the electrolyte used during the measurements plays a crucial role in the sensitive detection of arsenic. An exhaustive study of experimental conditions was addressed for the electrochemical fingerprinting of As(III) for the development of a fast and disposable sensor. The electrochemical detection was achieved after optimization of cathodic pre-concentration and stripping parameters by SWASV at modified SPCEs, proving its applicability for disposable and cost-effective in situ analysis of arsenic. Nevertheless, the overall sensor fabrication and analysis time was 5 min for Au-PtNPs/SPCE platform, and 12 min for Au-PtNPs/PANI/SPCE platform, respectively, allowing the As(III) detection in nanomolar range.

## 2. Materials and Methods

### 2.1. Reagents and Instrumentation

All chemicals were of analytical grade and all solutions were prepared in deionized water from a Mili-Q System (Milipore, Italy, 18 MΩ·cm). Lithium chloride (LiCl), sodium arsenite (NaAsO_2_), aniline (C_6_H_5_NH_2_), and hydrochloric acid 30% (HCl) were purchased from Merck (Darmstadt, Germany). Potassium chloride (KCl), potassium ferrocyanide (K_4_[Fe(CN)_6_]), potassium ferricyanide (K_3_[Fe(CN)_6_]), sulphuric acid 97% (H_2_SO_4_), perchloric acid (HClO_4_), tetrachloroauric acid (HAuCl_4_), hexachloroplatinic acid (H_2_PtCl_6_) were purchased from Sigma-Aldrich. Heavy metal solutions were prepared from Cu(II), Pb(II), and Hg(II) standard solutions of AAS grade purchased from Fluka (Cinisello Balsamo, Milan, Italy).

All electrochemical measurements were performed on an Autolab potentiostat *PGSTAT302*, using NOVA software 1.10 and 1.11. Screen-printed carbon electrochemical cells (SPCE) with a 3 mm diameter carbon working electrode, Ag/AgCl pseudo-reference electrode, and carbon as a counter electrode were purchased from EcoBioServices (Sesto Fiorentino, Florence, Italy).

### 2.2. Preparation of Composite Platforms

Nanocomposite modified electrodes were obtained by electropolymerization of aniline and electrodeposition of AuNPs, PtNPs and mixtures of these from their metallic salts (HAuCl_4_ and H_2_PtCl_6_). Aniline was electropolymerized by cyclic voltammetry (CV) through a procedure previously described [12,18]. In brief, 50 µL of 2.5 mM C_6_H_5_NH_2_ in 50 mM HClO_4_ solution was drop casted onto SPCE. The potential was scanned from –0.4 V to +0.8 V for 10 cycles at 50 mV s^−1^ scan rate. The electrodeposition of nanoparticles was adapted for SPCE from a method previously described [22]. AuNPs and PtNPs were electrodeposited at SPCE and PANI/SPCE by applying a fixed potential of −0.2 V for 130 s from solutions containing:

- 0.2 mM HAuCl_4_ in 0.5 M H_2_SO_4_;
- 2 mM HAuCl_4_ in 0.5 M H_2_SO_4_;
- 4 µM H_2_PtCl_6_ in 0.5 M H_2_SO_4_;
- 1 mM H_2_PtCl_6_ in 0.5 M H_2_SO_4_;
- and combination of these.

Electrochemical characterization of the developed platforms by cyclic voltammetry measurements was performed in a 5 mM [Fe(CN_6_)]^4−/3−^ in 0.1 M KCl solution at different rates (25–150 mV s^−1^), using a potential range from −0.50 to +0.80 V and a potential step of 4 mV.

### 2.3. Measurement Procedure

SWASV was performed in different electrolytes like (i) lithium chloride (6.0 M LiCl in 0.1 M H_2_SO_4_), (ii) potassium chloride (0.1 M KCl solution containing 0.1 M HCl), and (iii) hydrochloric acid (0.1 M HCl) for the determination of arsenic from a 50 mM NaAsO_2_ aqueous solution which was diluted to the desired concentrations using the electrolyte chosen for the experiment. SWASV conditions were: conditioning potential (*E_cond_*) +0.70 V for 30 s, cathodic deposition potential (*E_dep_*) −0.5 V for (*t_dep_*) 60 s, and equilibration time (*t_eq_*) of 30 s; and stripping parameters: 45 mV s^−1^ scan rate, 0.003 V step potential, and 15 Hz frequency (f). The measurements were performed immersing the modified electrochemical cell in a 5.0 mL solution. Stirring conditions were used during the accumulation step for a more uniformly deposition using a Metrohm 728 stirrer. Cathodic pre-concentration parameters (*E_dep_* and *t_dep_*) were optimized and the best electrolyte solution was chosen among these experiments.

## 3. Results and Discussion

In a first attempt, different types of modified screen-printed electrodes were tested in order to obtain the best analytical signal of As(III) electrochemical oxidation of pre-concentrated As(0) with minimum of modification steps. The SPCEs were preferred due to their possibility of single use, excluding thus the contamination issues and allowing in a future step, the integration into a decentralized analytical device.

### 3.1. Electrochemical Characterization of the Platform Configurations

**Figure 1:**
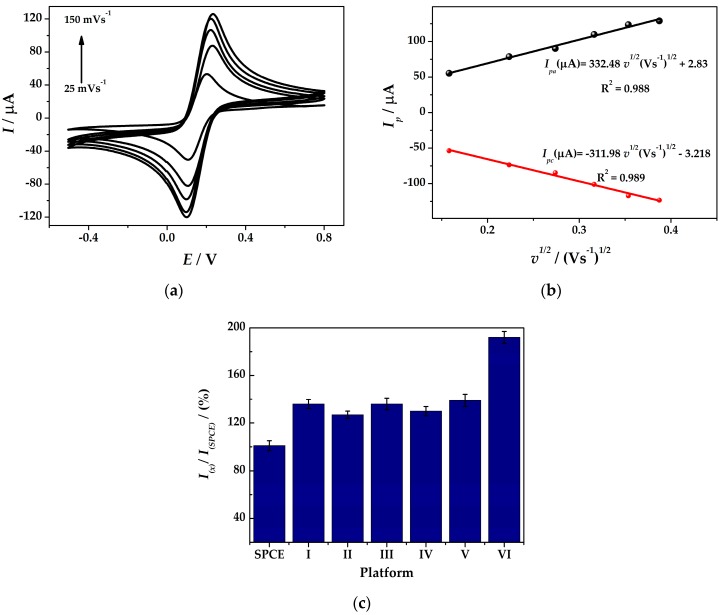
(**a**) CVs in 5 mM [Fe(CN_6_)]^4–/3–^ in 0.1 M KCl solution at (**IV**)Au-PtNPs/SPCE at rates of 25–150 mV s^−1^ and (**b**) plots of anodic and cathodic peak currents versus square root of scan rate; (**c**) electroactive surface area % versus geometric area of a bare SPCE compared to SPCE modified with: (**I**)AuNPs, (**II**)PtNPs^i^, (**III**) PtNPs^ii^, (**IV**)Au-PtNPs, (**V**) PANI, and (**VI**)Au-PtNPs/PANI.

Unmodified and modified SPCE with different nanostructured films: gold nanoparticles (AuNPs/AuSPCE), platinum nanoparticles (PtNPs/SPCE), bimetallic (Au-PtNPs/SPCE), polymer (PANI/SPCE), and composite (Au-PtNPs/PANI/SPCE) were characterized by cyclic voltammetry (CV) in 5 mM [Fe(CN_6_)]^4−/3−^ in 0.1 M KCl solution. Table 1 shows the investigated electrochemical platform architectures. Choosing the best platform for further optimization in order to develop the sensor for As(III) fingerprinting, different parameters were taken into consideration. Hence, beside the electrocatalytic effect, the efficiency of the electrode design in terms of reproducibility, costs and fabrication time of the platforms towards arsenic detection were also evaluated. The platforms that offered the best results, regarding the above description, are represented in Figure 1c.

Therein, the effective surface area of all six platform designs were estimated by CV following Randles–Sevcik Equation (1) [23]:

*I*_p_ = (2.69 × 10^5^)*n*^3/2^*AD*^1/2^*v*^1/2^*C* (1)

where *I*_p_ is the response peak current (A), *n* is the electrons transfer number, *A* is the electrochemical effective area (cm^2^), *D* is the diffusion constant of 5.0 mM K_3_Fe(CN)_6_ in 0.1 M KCl (6.2 × 10^−6^ cm^2^ s^−1^), *v* is the scan rate (V s^−1^), and *C* relates to the bulk concentration of the K_3_Fe(CN)_6_ redox probe (mol cm^−3^).

Based on the above formula, the electrochemical effective area can be calculated from the slope of the *I*_p_-*v*^1/2^ relations. Figure 1a,b presents the CVs of 5.0 mM K_3_Fe(CN)_6_ in 0.1 M KCl solution registered at (**IV**)Au-PtNPs/SPCE at scan rates between 25 and 150 mV s^−1^ and the linear plots of the anodic and cathodic peak currents versus square root of scan rate, respectively. The electrochemical surface area of the platforms was calculated and compared with the one of bare SPCE (%/bare SPCE geometric area).

It can be seen from Figure 1c that the effective surface area of platform (**I**)AuNPs/SPCE increases with a factor of 1.33 compared with bare SPCE. Moreover, two platform architectures were evaluated for the electrodeposition of PtNPs at SPCE with respect to the H_2_PtCl_6_ solution concentration. It can be clearly seen that the use of a more concentrated chloroplatinic acid solution is not pertinent as the electrocatalytic effect towards the redox probe is approximately the same for (**II**)PtNPs/SPCE (127%) and (**III**)PtNPs/SPCE (130%), respectively. Hence, when the bimetallic platform (**IV**)Au-PtNPs was designed, a slightly higher effective surface area was obtained (136%) and, in particular, when in combination with PANI, amplified peak currents and more reversible redox peaks were obtained, which were related to around a two-fold increase in the electroactive surface area of (**VI**)Au-PtNPs/PANI/SPCE (192%) compared to bare SPCE. The synergistic effect of the nanocomposite hybrid film could be observed as (**V**)PANI/SPCE exhibited only an increase of the electrode surface area of 139% compared to bare SPCE, thus proving improved electrocatalytic effect of platform (**VI**). Moreover, the time needed for platform development should be considered to provide an efficient and rapid sensor. The metal nanoparticles-modified SPCE fabrication involves a constant potential assisted electrodeposition for 130 s, while electropolymerization of aniline at SPCE occurs after 480 s. All results are reported in triplicate with a RSD% lower than 5%. Therefore, overall characteristics of the developed platforms were considered when applied for arsenic fingerprinting. AuNPs and PtNPs are excellent electrical conductors and their incorporation in the sensor’s design improves the electron transfer during the electrochemical processes. In addition, it was demonstrated that the Pt sites work as atomic hydrogen and molecular hydrogen generator in H_2_SO_4_ solution which can chemically reduce the As(III) to As(0) and, therefore, enhance the cathodic pre-concentration of As(0) at both the Pt sites and the neighboring Au sites [22]. Therefore, the platforms with the best analytical performance were chosen for further experiments namely (**IV**)Au-PtNPs/SPCE and (**VI**)Au-PtNPs/PANI/SPCE, respectively.

### 3.2. Optimization of the Experimental Conditions

**Figure 2:**
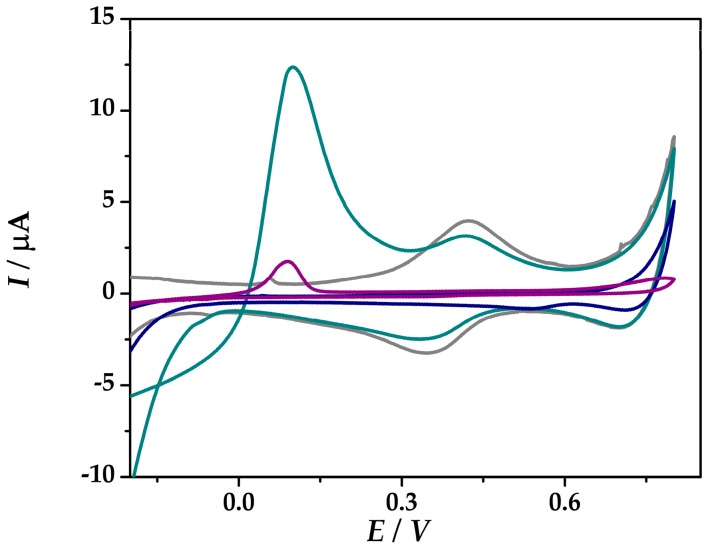
CVs recorded at (**IV**)Au-PtNPs/SPCE in 0.1 M HCl (blue), in the presence of 1 mM As(III) in 0.1 M HCl (purple), and at (**VI**)Au-PtNPs/PANI/SPCE in 0.1 M HCl (grey), in the presence of 1 mM As(III) in 0.1 M HCl (dark cyan) at 50 mV s^−1^.

Figure 2 shows the cyclic voltammograms of the developed platforms (**IV**) and (**VI**) in 0.1 M HCl and in the presence of 1 mM As(III) in 0.1 M HCl without any prior pre-concentration step. No redox peaks were found in 0.1 M HCl (iii) free of As (III) at Au-PtNPs/SPCE in the potential range from −0.5 V and +0.8 V, while at Au-PtNPs/PANI/SPCE the redox pair at around 0.38 V was assigned to the oxidation/reduction of PANI. Both platforms show a peak at around 0.1 V, which is assigned to the electrooxidation of As(0). At (**VI**)Au-PtNPs/PANI/SPCE, the peak current intensity was 11.6 µA, while at (**IV**)Au-PtNPs/SPCE lower current intensity was obtained (1.94 µA), thus proving an enhanced sensitivity of As(III) at platform (**VI**).

The methods involving a potential-assisted electrochemical pre-concentration for trace metals sensing continues to be one of the most powerful techniques for heavy metal detection. Therefore, SWASV measurements were employed for As(III) sensing at the developed nanostructured platforms in order to obtain improved sensitivities. The mechanism for the enhanced SWASV signaling of As(III) involves a cathodic pre-concentration step of As(0) from a As(III) solution followed by the anodic stripping from the electrode surface characteristic for the re-oxidation of As(0) to As(III). Therefore, the magnitude of the electrooxidation peak was used for the quantitative evaluation of As(III).

In order to obtain the best results for As(III) fingerprinting, several electrolytes (6.0 M LiCl in 0.1 M H_2_SO_4_ (i), 0.1 M KCl in HCl (ii), and 0.1 M HCl (iii)) were tested for the optimized designed platforms both in the presence and the absence of As(III). Several parameters like the As(III) deposition (pre-concentration) time (*t_dep_*) and potential (*E_dep_*), electrochemical method, the response and the linearity of the developed platforms towards As(III) were studied and optimized for all electrolyte solutions.

**Figure 3:**
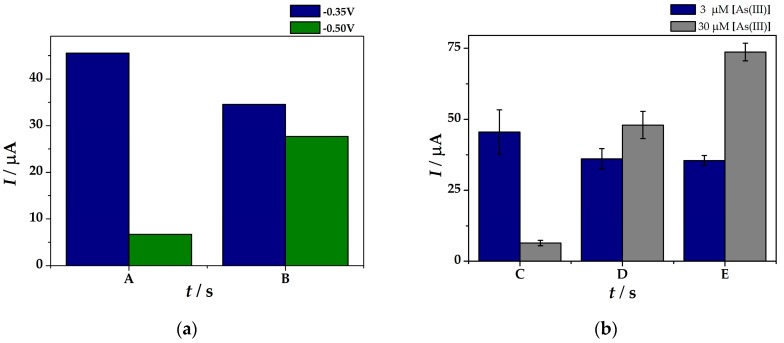
Peak current intensities obtained by means of SWASV measurements at (**IV**)Au-PtNPs/SPCE from solutions containing 3 µM As(III) (blue) and 30 µM As(III) (grey) in 6.0 M LiCl in H_2_SO_4_ 0.1 M by potential-assisted cathodic pre-concentration of (**a**) *t_dep_* = 120 s (A) and 240 s (B) at E*_dep_* = −0.35 V (blue) and E*_dep_* = −0.50 V (green); and (**b**) *t_dep_* = 120 s (C), 180 s (D), 240 s (E) at E*_dep_* = −0.50 V.

Firstly, the fingerprinting of As(III) was assessed in electrolyte (i). Figure 3a shows the peak currents intensities obtained in a solution containing 3 µM As(III) in 6.0 M LiCl in 0.1 M H_2_SO_4_ (**i**) at (**IV**)Au-PtNPs/SPCE where the SWASV parameters were optimized taking into account two cathodic pre-concentration times of 120 s (A) and 240 s (B) and potentials of −0.35 V and −0.50 V, respectively. Foremost, the influence of the pre-concentration time was considered. It can be observed that at *t_dep_* = 120 s pre-concentration time there is a great difference in the peak current values when applying *E_dep_* = −0.35 V (45.54 µA) compared to *E_dep_* = −0.5 V (6.66 µA). For longer cathodic pre-concentration time (*t_dep_* = 240 s), an increase in the peak current intensity (27.64 µA) was obtained for *E_dep_* = −0.5 V, whereas for *E_dep_* = −0.35 V a slightly decrease down to 34.54 µA was observed. Although higher peak current intensities were obtained at –0.35 V pre-concentration applied potential. When increasing the deposition time, the results showed lower current intensities for higher As(III) levels. Moreover, the results showed better %RSD values when applying lower deposition potential (*E_dep_* = −0.5 V).

Secondly, for a better understanding of the cathodic pre-concentration step, two arsenic concentration solutions were tested, namely 3 µM and 30 µM As(III) in electrolyte solution (**i**). Therefore, in order to increase the sensitivity of the sensor, different cathodic pre-concentration deposition time was considered (120 s, 180 s, and 240 s).

At *t_dep_* = 120 s, irrelevant results in arsenic anodic stripping were obtained as the peak intensity of the more concentrated As(III) solution revealed lower current values due to a probably low sensitivity at higher concentration levels. When increasing the pre-concentration time up to 180 s, better analytical response of 36.1 µA and 48.0 µA were obtained at modified electrodes for 3 µM and 30 µM As(III) solution, respectively. The results demonstrate higher electrocatalytic effect of the (**IV**)Au-PtNPs/SPCE platform when the potential was applied for *t_dep_* = 240 s by comparing the peak current intensity ratio (a two-fold increase) obtained in 3 µM and 30 µM As(III) solution (Figure 3b). However, high cathodic pre-concentration time could lead to irreproducible results as difficulties in stripping the As(0) from the electrode surface could be encountered, leading thus to a decrease of the sensors’ reproducibility in terms of %RSD. Therefore, different strategies should be followed for a more sensitive detection of arsenic.

The effect of HAuCl_4_ solution concentration was studied for this supporting electrolyte, using 2 mM and a 0.2 mM solutions of HAuCl_4_ in the HAuCl_4_-H_2_PtCl_6_ mixture and testing by SWASV measurements in a 30 µM As(III) in electrolyte solution (i). By increasing the HAuCl_4_ concentration and the Au-PtNPs ratio from the sensor structure, higher oxidation peak current intensities of As(III) were obtained, allowing the detection of trace levels of As(III). Further experiments were performed using platform number (**IV**) obtained by electrochemical deposition of Au-PtNPs at SPCE from a mixture containing 2 mM HAuCl_4_ and 4 µM H_2_PtCl_6_ in 0.5 M H_2_SO_4_ solution.

In order to increase both sensitivity and reproducibility of the sensors compared with the results obtained in electrolyte (i), another electrolyte solution was used, namely 0.1 M KCl in 0.1 M (ii).

**Figure 4:**
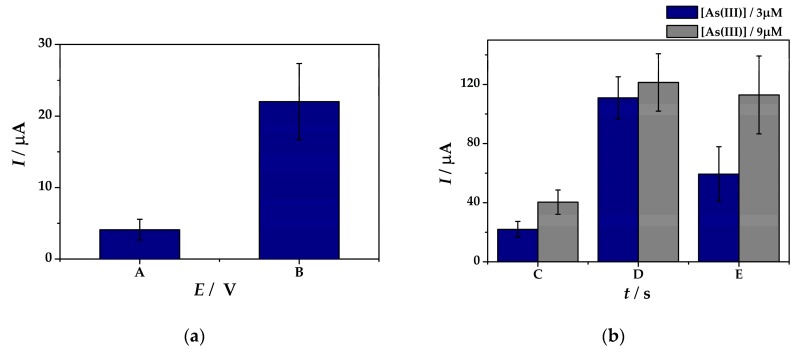
Peak current intensities obtained by means of SWASV measurements at (**IV**)Au-PtNPs/SPCE from solutions containing 3 µM As(III) (blue) and 9 µM As(III) (grey) in 0.1 M KCl in 0.1 M HCl, (ii) using a cathodic pre-concentration (**a**) potential (*E_dep_*) of −0.35 V (A) and −0.50 V (B) at *t_dep_* = 120 s; and (**b**) time (*t_dep_*) of 120 s (C), 180 s (D), 240 s (E) at E*_dep_* = −0.50 V.

Figure 4a shows the peak current intensities recorded by SWASV at (**IV**)Au-PtNPs/SPCE from a 3 µM As(III) solution in electrolyte (ii) using a *t_dep_* = 120 s at two different deposition potentials *E_dep_*: −0.35 V and −0.5 V. The higher peak height was obtained after the deposition of As(0) from a As(III) solution at −0.50 V potential (22 µA) compared to −0.35 V (4.11 µA). Therefore, −0.50 V cathodic pre-concentration potential of As(0) was used for further optimization tests.

In the next step, the cathodic potential-assisted deposition time was optimized as it plays an important role in the sensitivity and reproducibility of the obtained sensors (Figure 4b). The electrochemical experiments were conducted at (**IV**)Au-PtNPs/SPCE in solutions containing 3 µM and 9 µM of As(III), respectively. The parameters of the cathodic pre-concentration of As(0) were *E_dep_* of –0.50 V and *t_dep_* of 120, 180, and 240 s, respectively. Increased values of the As(0) anodic stripping peaks are observed in correlation with the increase in the deposition time from 120 s to 180 s from a solution containing 3 µM As(III). Therefore, after cathodic pre-concentration of *t_dep_* = 120 s a peak current of 22.0 µA was obtained, whereas after *t_dep_* = 180 s the peak current height was 110.95 µA, respectively. By contrast, when comparing the results obtained in 9 µM As(III) solution, little changes in the peak current values were observed after 180 s cathodic pre-concentration (121.35 µA), thus proving the low sensitivity of the sensor. When applying 240 s deposition time show an increase in the peak currents up to 59.35 µA and 112.92 µA from solutions of 3 µM As(III) and 9 µM As(III) solution in 0.1M KCl in 0.1M HCl, respectively. Although the results show an increase in the sensitivity of the sensor compared to the one using 120 s deposition time, high %RSD values of 18% and 26% were obtained at (**IV**)Au-PtNPs/SPCE from solutions containing 3 µM As(III) (blue) and 9 µM As(III) (grey) in 0.1 M KCl in 0.1 M HCl, thus proving its low reproducibility.

As could be seen from the previous experiments the As(III) fingerprint was assessed in two different supporting electrolytes (i and ii). It can be concluded that –0.5 V cathodic pre-concentration potential showed the best results in both electrolytes to reduce the As(III) to As(0) at the electrode surface. The time of the potential-assisted deposition was evaluated. Although improved sensitivities were obtained when at *t_dep_* = 240 s in electrolytes (i) and (ii), the relatively high %RSD values and long analysis time reduce the utility for future possible in situ analysis.

The influence of the KCl salt was also considered, thus the optimization of the electrocatalytic effect of the (**IV**)Au-PtNPs/SPCE platform towards arsenic was evaluated as well in 0.1 M HCl supporting electrolyte (iii) free of KCl.

**Figure 5:**
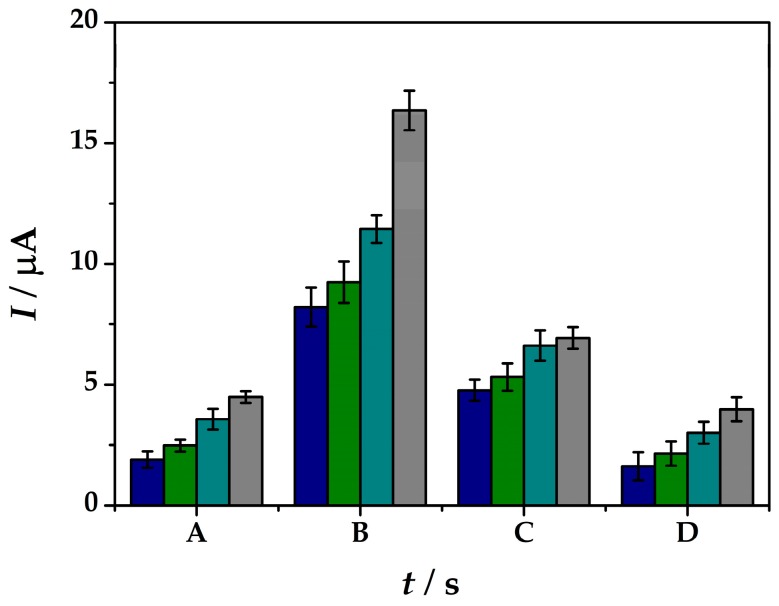
Peak current intensities obtained by means of SWASV measurements at (**IV**)Au-PtNPs/SPCE from solutions containing As(III) of 33 nM (blue), 67 nM (green), 133 nM (cyan), 200 nM (grey) in 0.1 M HCl (iii) by applying a cathodic pre-concentration time (*t_dep_*) of 60 s (A), 120 s (B), 180 s (C), 240 s (D) at *E_dep_* = −0.50 V.

In this case different concentrations of the As(III) solutions were tested in the range between 33–200 nM in order to see the influence of the pre-concentration time for As(III) detection. It can be observed from Figure 5 not only the inversely correlation between the peak current intensities obtained within the SWASV tests with the arsenic cathodic pre-concentration period at the platform surface, but also the linear correlation with the As(III) concentration solutions. At *t_dep_* = 60 s of pre-concentration potential-assisted deposition of As(III) at the modified electrode surface shows peak current intensities from 1.90 µA at 33 nM to 4.49 µA for 200 nM As(III) solutions in electrolyte (iii). Although, 120 s of deposition offers the best current intensity increase from 8.21 µA at 33 nM to 16.34 µA for 200 nM As(III), the increase does not follow a linear correlation with the As(III) concentration. At 180 s deposition time lower current intensities were obtained and the linear correlation within the arsenic concentration was lost. For 240 s deposition time of As(0) the current intensities drop off to lower values as 1.62 µA at 33 nM to 3.98 µA for 200 nM As(III) solutions in electrolyte (iii). Therefore, considering the ratio between the sensitivity offered by the sensor and the analysis time needed, *t_dep_* = 60 s and *E_dep_* = −0.5 V in 0.1 M HCl solution were used as a cathodic pre-concentration step during the SWASV analysis method at (**IV**)Au-PtNPs/SPCE platform.

Finally, after all optimization steps made for each electrolyte solution, an overall comparison is presented taking into consideration the oxidation peak current intensities obtained at platform (**IV**) in a solution containing 3 µM As(III) in 6.0 M LiCl in 0.5 M H_2_SO_4_ (i), 0.1 M KCl in 0.1 M HCl (ii), and 0.1 M HCl (iii)**,** respectively. Firstly, As(III) was electrochemically reduced by potential-assisted pre-concentration using at *E_dep_* of −0.50 V for 120 s. Platform (**IV**) revealed the highest electrocatalytic effect against As (6.66 µA) in electrolyte (i), compared with the SWASV oxidation peaks values of 5.07 µA and 2.76 µA obtained in electrolyte (ii) and (iii), respectively.

At trace levels of arsenic, the sensitivities of the sensor is considerably reduced in electrolytes (i) and (ii) compared to the one obtained in electrolyte (iii). The fact that these electrolytes offers a peak only at high levels of As(III) makes the electrolyte (iii) the best solution for sensing As(III) at the developed platform. Nevertheless, the overall sensor fabrication and analysis time was 5 min for platform (**IV**)Au-PtNPs/SPCE, and 12 min for platform (**VI**)Au-PtNPs/PANI/SPCE, respectively; thus, proving its high efficiency as a possible future disposable sensor for arsenic sensing.

### 3.3. Arsenic Electrochemical Fingerprint Detection

The electrochemical detection of arsenic was performed at platforms (**IV**)Au-PtNPs/SPCE and (**VI**)Au-PtNPs/PANI/SPCE using the optimized square wave anodic stripping voltammetry procedure as previously reported. Different concentration solutions of As(III) (33–200 nM) in a 0.1 M HCl (iii) solution were tested using SWASV by applying a pre-concentration deposition potential of −0.50 V for 60 s in the potential range from −0.20 to +0.50 V at 45 mV s^−1^.

**Figure 6:**
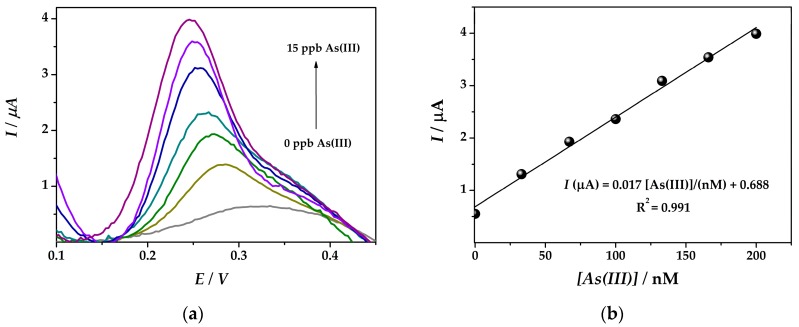
(**a**) SWASV obtained at (**IV**)Au-PtNPs/SPCE platform (*E_dep_* = −0.5 V; *t_dep_* = 60 s) with arsenic solution concentrations of 33, 67, 100, 133, 166, 200 nM As(III) in 0.1 M HCl solution and (**b**) the corresponding calibration plot.

A linear correlation between the arsenic concentration and the signal obtained by SWASV in 0.1 M HCl at bimetallic nanocomposite electrode surface (**IV**) was obtained between 33 nM and 200 nM As(III) with a sensitivity of 0.017 µA nM^−1^ and a limit of detection (LOD) of 19.7 nM (Figure 6). The detection limit was calculated from three times the standard deviation of the regression equation divided by its slope [23].

Hence, the response of platform (**VI**)Au-PtNPs/PANI/SPCE was evaluated in the optimized conditions for arsenic detection as revealed the highest electrocatalytic effect towards 5 mM [Fe(CN_6_)]^4−/3−^ in 0.1 M KCl solution and by CV characterization in 1 mM As(III) in electrolyte (i). By contrast, when applied for arsenic sensing in trace levels, platform (**VI**) exhibited lower current intensities compared to platform (**IV**) and offered a linearity in the range between 0.1–1.25 mM As(III) with the following equation *I*(µA) = 6.116 [As(III)/mM] + 6.129; R^2^ = 0.994. This could be probably due to the incorporation of the deposited As(0) into the PANI matrix and lower catalytic effect of the electrogenerated noble metal particles. Moreover, the Au-Pt sites could be entrapped into the polymer film and therefore the enhanced cathodic pre-concentration would not occur. Although, for high levels of As(III), (**VI**)Au-PtNPs/PANI/SPCE could be applied.

Both platforms could be used for fingerprinting As(III) in 0.1 M HCl solution, where (**IV**) provided better responses for low levels of As(III), meanwhile the platform containing PANI (**VI**) could be useful for detecting As(III) levels in milimolar range.

### 3.4. Interferences

As(III) is rarely found in water or sand by itself. Between all the possible interference metals, that can be found alongside As(III), Cu(II), Pb(II), and Hg(II) are very common and can easily interfere in the detection of the desired metal.

SWASVs were performed at platform (**IV**) in different solutions containing 3 µM Hg(II), Cu(II), Pb(II), and As(III) in 0.1 M HCl using the previously optimized method. In addition to the specific oxidation peak of As(0) to As(III) at +0.20 V, a peak separation was observed when in combination with Hg(II) (+0.25 V) and Cu(II) (+0.26 V) interferences. Nevertheless, even the signals of Cu(II) and Hg(II) are in a close potential range to the one of As(III), and their current intensities are much lower (Table 2).

It was thus shown that using the optimized (**IV**)Au-PtNPs/SPCE platform it was possible to detect As(III) even in the presence of other metallic ions.

## 4. Conclusions

A novel and easy to use nanohybrid platform based on a mixed film of PANI and bimetallic composite Au-PtNPs for As(III) electrochemical fingerprinting was optimized. In addition to many advantages of PANI, like the fast synthesis, low cost, and high electrochemical conductivity, to overcome its drawbacks PANI was used in hybrid platforms with Au-PtNPs. In order to optimize a sensor for As(III) detection in water several electrolytes were evaluated in order to obtain the best analytical performance for As sensing. The electrochemical detection with a good limit of detection was achieved after optimization of cathodic pre-concentration and stripping parameters by square wave anodic stripping voltammetry at modified screen-printed carbon-based electrochemical cells. The sensors allowed the As(III) determination in the range between 0 and 200 nM As(III) and a LOD of 19.7 nM was obtained. The platforms with the best analytical performance were Au-PtNPs/SPCE and Au-PtNPs/PANI/SPCE. Hence, first the platform revealed better sensitivity in solutions of trace levels of As(III), while the second platform offered better results for higher concentrations of As(III). Nevertheless, the overall sensor fabrication and analysis time was 5 min for platform Au-PtNPs/SPCE, and 12 min for platform Au-PtNPs/PANI/SPCE, respectively. These preliminary studies prove the practical applicability and high efficiency as possible future disposable and cost-effective sensors for in situ arsenic sensing.

## Figures and Tables

**Table 1:** Platform electrode configuration used in the study.

Platform Design		Experimental Conditions	
		Solution Concentration	Electrodeposition Parameters
(**I**)	AuNPs/SPCE	2 mM HAuCl_4_ in 0.5 M H_2_SO_4_	−0.20 V for 130 s
(**II**)	PtNPs^i^/SPCE	4 µM H_2_PtCl_6_ in 0.5 M H_2_SO_4_	−0.20 V for 130 s
(**III**)	PtNPs^ii^/SPCE	1 mM H_2_PtCl_6_ in 0.5 M H_2_SO_4_	−0.20V for 130 s
(**IV**)	Au-PtNPs/SPCE	Mixture of 2 mM HAuCl_4_ and 4 µM H_2_PtCl_6_ in 0.5 M H_2_SO_4_	−0.20 V for 130 s
(**V**)	PANI/SPCE	2.5 mM C_6_H_5_NH_2_ in 50 mM HClO_4_;	CV: −0.40–0.80 V vs. Ag/AgCl, 10 scans, 50 mV s^−1^ at SPCE
(**VI**)	Au-PtNPs/PANI/SPCE	2.5 mM C_6_H_5_NH_2_ in 50 mM HClO_4_; Mixture of 2 mM HAuCl_4_ and 4 µM H_2_PtCl_6_ in 0.5 M H_2_SO_4_	CV: −0.40– 0.80 V vs Ag/AgCl, 10 scans, 50 mV s^−1^ at SPCE −0.20 V for 130 s at PANI/SPCE

**Table 2:** SWASV signal for As(III), Cu(II), Pb(II), Hg(II) solution in 0.1 M HCl in the potential range from 0 to +0.6 V; *t_dep_* = 60s, *E_dep_* = −0.50 V.

Metal	E/V	I/µA
As(III)	+0.20	8.93
Cu(II)	+0.26	0.96
Pb(II)	+0.47	5.67
Hg(II)	+0.25	1.87

## References

[1] Cinti S., Politi S., Moscone D., Palleschi G., Arduini F. (2014). Stripping Analysis of As(III) by means of screen-printed electrodes modified with gold nanoparticles and carbon black nanocomposite. Electroanalysis.

[2] Roy P., Saha A. (2002). Metabolism and toxicity of arsenic: A human carcinogen Sources of different forms of arsenic: Human exposure and chronic arsenicism. Curr. Sci..

[3] National Research Council (U.S.) Subcommittee on Arsenic in Drinking Water. https://www.who.int/news-room/fact-sheets/detail/arsenic.

[4] D’Ippoliti D., Santelli E., De Sario M., Scortichini M., Davoli M., Michelozzi P. (2015). Arsenic in drinking water and mortality for cancer and chronic diseases in Central Italy, 1990–2010. PLoS ONE.

[5] Barbieri M., Nigro A., Sappa G. (2014). Arsenic contamination in groundwater system of Viterbo area ( Central Italy). Senses Sci..

[6] Yogarajah N., Tsai S.S.H. (2015). Detection of trace arsenic in drinking water: Challenges and opportunities for microfluidics. Environ. Sci. Water Res. Technol..

[7] Chen J., Rosen B.P. (2014). Biosensors for inorganic and organic arsenicals. Biosensors.

[8] Kempahanumakkagari S., Deep A., Kim K.H., Kumar Kailasa S., Yoon H.O. (2017). Nanomaterial-based electrochemical sensors for arsenic—A review. Biosens. Bioelectron..

[9] Hung D.Q., Nekrassova O., Compton R.G. (2004). Analytical methods for inorganic arsenic in water: A review. Talanta.

[10] Guo Z., Yang M., Huang X.-J. (2017). Recent developments in electrochemical determination of arsenic. Curr. Opin. Electrochem..

[11] Liu Z.G., Huang X.J. (2014). Voltammetric determination of inorganic arsenic. TrAC—Trends Anal. Chem..

[12] Rapini R., Marrazza G. (2017). Electrochemical aptasensors for contaminants detection in food and environment: Recent advances. Bioelectrochemistry.

[13] Henze G., Wagner W., Sander S. (1997). Speciation of arsenic(V) and arsenic(III) by cathodic stripping voltammetry in fresh water samples. Fresenius. J. Anal. Chem..

[14] Compton R.G., Rassaei L., Sillanpää M., Marken F., French R.W. (2008). Arsenite Determination in Phosphate Media at Electroaggregated Gold Nanoparticle Deposits. Electroanalysis.

[15] Chowdhury A.-N., Ferdousi S., Islam M.M., Okajima T., Ohsaka T. (2007). Arsenic detection by nanogold/conducting-polymer-modified glassy carbon electrodes. J. Appl. Polym. Sci..

[16] Dai X., Compton R.G. (2006). Detection of As(III) via oxidation to As(v) using platinum nanoparticle modified glassy carbon electrodes: Arsenic detection without interference from copper. Analyst.

[17] Selvolini G., Băjan I., Hosu O., Cristea C., Săndulescu R., Marrazza G. (2018). DNA-based sensor for the detection of an organophosphorus pesticide: Profenofos. Sensors (Switzerland).

[18] Saberi R.S., Shahrokhian S., Marrazza G. (2013). Amplified electrochemical DNA sensor based on polyaniline film and gold nanoparticles. Electroanalysis.

[19] Ravalli A., Rossi C., Marrazza G. (2017). Bio-inspired fish robot based on chemical sensors. Sensors Actuators B Chem..

[20] Rapini R., Cincinelli A., Marrazza G. (2016). Acetamiprid multidetection by disposable electrochemical DNA aptasensor. Talanta.

[21] Ma J., Tao X.Y., Zhou S.X., Song X.Z., Lin-Guo, Yao-Wang, Zhu Y.B., Guo L.T., Liu Z.S., Fan H.L. (2019). Facile fabrication of Ag/PANI/g-C 3 N 4 composite with enhanced electrochemical performance as supercapacitor electrode. J. Electroanal. Chem..

[22] Bu L., Liu J., Xie Q., Yao S. (2015). Anodic stripping voltammetric analysis of trace arsenic(III) enhanced by mild hydrogen-evolution at a bimetallic Au-Pt nanoparticle modified glassy carbon electrode. Electrochem. Commun..

[23] Brett C.M.A., Oliveria Brett A.M. (1993). Electrochemistry principles methods and applications.

